# The calculated responses against COVID-19 in Namibia

**DOI:** 10.11604/pamj.supp.2020.37.1.25697

**Published:** 2020-10-16

**Authors:** Josephine Ndapewoshali Amesho, Attaullah Ahmadi, Don Eliseo Lucero-Prisno III

**Affiliations:** 1 Ministry of Health and Social Services of Namibia, Windhoek, Namibia,; 2 Kabul University of Medical Sciences, Kabul, Afghanistan,; 3 Department of Global Health and Development, London School of Hygiene and Tropical Medicine, London, United Kingdom,; 4 Faculty of Management and Development Studies, University of the Philippines (Open University), Los Baños, Laguna, Philippines

**Keywords:** Coronavirus, COVID-19, pandemic, lockdown, responses, Namibia

## Abstract

COVID-19 has spread to many countries and infected a vast number of people around the world. Namibia is not spared from this disease. On the early days of the pandemic in Namibia, the government instituted a four-stage strategy--from a full lockdown in Stage One with gradual relaxation of restrictions in Stage Two and ending with Stage Four. This was proven to be effective since the number of daily new cases were minimal by the time the restrictions were lifted in Stage Four, which allowed many non-essential businesses to resume, and borders to reopen. However, following this, the cases jumped in an alarming pace. The situation was also exacerbated partly by obliviousness of the people to restrictions due to their economic issues. At present, the government struggles to bring the situation back under control. Thus, the government reintroduced new restrictions to mitigate the situation. The country is facing paucity of facilities like personal protective equipment (PPE), health workers, intensive care unit (ICU) equipment and testing kits. To avoid further explosion of cases, Namibia needs to determine relaxation of restrictions based on the indicators of the situation of COVID-19. Assistance to the population and addressing insufficiency of facilities by the government through innovative solutions are of utmost importance in tussling the virus.

## Commentary

The novel coronavirus (COVID-19) is a global public health complication [1] that has posed huge ramifications on humans since it started. Namibia like many other African countries, is ensnared with this plague. As of 22 August 2020, 5227 confirmed cases with 42 deaths and 2457 recoveries [2] in 12 of 14 regions have been reported. Erongo has the highest number of cases and indicates clustered community transmission, followed by Khomas [3]. The country is fourth in terms of rate of daily new cases in the continent after South Africa, Eswatini and Gabon [4]. There are only three testing laboratories operational in Namibia: National Institute of Pathology (NIP), Pathcare and University of Namibia (UNAM) [3]. Following WHO promulgation of COVID-19 as a pandemic, the Ministry of Health and Social Services, warned the whole nation on the consequences of underestimating COVID-19 contagion. The Namibian government has exerted efforts to prevent the spread of the virus, however, the cases have been increasing. This commentary elucidates the course of COVID-19 in Namibia as well as the measures taken by the government to contain the virus.

The first case of COVID-19 was recorded on 13^th^ March 2020. This was an imported case of a Romanian couple (a 35-year-old man and a 25-year-old woman) who travelled from Madrid Spain via Doha Qatar. Although they have been screened upon arrival as per protocol, it did not yield any alarming information. The man showed symptoms of cough and high fever, and the woman had only fever. Both tested positive later [3]. Both patients were kept in an isolation center for three months until they finally tested negative. As there had been cross infection and the patients did not observe social distancing between each other, hence their failure to recover quickly. Upon confirmation of the two first cases, like many other African countries [5], the government instituted a series of preventive measures to curb the spread of the virus. First, contact racing was started. All public gatherings were suspended for a period of 30 days. All outbound and inbound flights from and to Qatar, Germany and Ethiopia were banned [3]. On 24^th^ March, the government declared a state of emergency and the borders between Namibia and South Africa and other neighboring countries were immediately closed [6]. This was to allow supply of food and other essentials goods to Namibia. When the few confirmed cases started to appear, on 30^th^ April 2020 the government announced to impose stages of emergency depending on the situation--Stages One to Four with different degrees of lockdown, full lockdown under Stage One, gradual relaxation of restrictions under Stage Two and completing at the end of Stage Four [6]. Stage one was scheduled on 28 March to 4 May. All non-essential services were closed down including the prohibition of the sale of alcohol, closure of schools and churches and gyms. Street vendors were not allowed to sell anything and all clothing stores were banned. All gatherings including weddings and funerals were only allowed to be carried out among a group of 10 people in maximum. Essential workers were allowed out on the streets while the rest of the people were recommended to stay home. People were required to do all meetings online. Only a few pharmacies and grocery shops were allowed to open according to a set schedule. The Ministry of Health and Social Services created a Rapid Response Team which consisted of permanent staff as well as a roster of all medical personnel to attend to wherever the need was, which was mainly in the capital city as the other towns had zero cases of COVID19 at that time [6].

The government pronounced travel restrictions into and out of the country excluding all repatriation travels. All of the people returning home had to be kept in a 14-day mandatory quarantine. They were swabbed upon arrival and on day 14, before being released. Restrictions on all travels within the country were also imposed. Only absolutely necessary ones, for instance, funerals or emergency medical conditions were permitted. All medical referrals to the major hospitals in the capital city were allowed to use transportation if the situation was an emergency [6]. Social mobilization and awareness raising was mounted through local media (TV, radio, print and electronics) so everyone had access to information. The Ministry of Health and Social Services also opened a toll free line anyone with symptoms could call [6]. For the country´s preparedness, a team of doctors and nurses as well as the new graduates were mobilized to ensure there was a doctor who attended to the sick patients in isolation as well as swabbing of the new arriving people. A specific center for swabbing was established in the capital city, Windhoek, for all suspected cases [6]. As a response to the pandemic, the government rented out different facilities for isolation and quarantine of everyone arriving in the country. The main testing center was also treated as a ward for suspected cases with medical conditions to be attended to. Currently, a section of one of two of the state hospitals in Windhoek has been closed off and converted into a self-contained intensive care unit (ICU) for patients, and admitted any positive case requiring care. The COVID-19 ICU also has a theatre that caters for any type of surgery including maternity cases. Initially the testing policy was only for patients who met the criteria for COVID-19 and traced for contacts with previous confirmed cases. The guideline has since changed and all patients presenting with anosmia are candidates for testing. There are no measures for mass testing in place yet [6]. In this stage the community was quite alarmed about the spread of the virus and was very much engaged and followed the rules as stipulated, except for those living in the rural areas. These rural residents were running their daily routine as usual as their lives depended on their daily sales of fresh produce and trying to sell other handmade goods.

Restrictions were lifted in Stage Two (Reopen with Strict Precautions) on 5 May as scheduled [3]. Travels between the cities, towns and regions within the country, and productive operations of all sectors were resumed with the condition that they observe social distancing guidelines. Opening of shopping mall/ retail outlets, restaurants, hairdressers, barbers and tailors and laundromats was subject to conditions [6]. Stage Three (Reopen with Moderate Precautions) lasted from 1^st^ June to 29^th^ June, in all regions except Walvis Bay Local Authority Area. Due to two recent positive cases, this area was reverted to Stage One [3]. This stage allowed high risk activities like theaters, cinemas, gyms, exercise centers, contact sports and sport events, gambling centers, nightclubs, concerts, workshop, summits, seminars and conferences, but subject to social distancing and hygiene protocols. Alcohol was only allowed for takeaway and private consumption [6]. On 29 June, the country entered Stage Four (The New Normal), except for Walvis Bay, Swakopmund, and Arandis Local Authority Areas in Erongo which remained in Stage Three (extended until 31 August 2020) due to the deterioration of the situation. In this stage, gradual reopening of borders to selective countries started while observing the guidelines. 250 are allowed to gather in public. People entering the country must show a 72-hour COVID-19 negative test result [3]. Resumption of normal flights, educational institutions, alcohol consumption in bars and restaurants were allowed in this stage [6].

This trajectory of the COVID-19 pandemic has tremendously crippled the already stressed Namibian economy. According to the Consolidated Approach for Reporting Food Insecurity Indicators (CARI), 36 per cent of the total population-nearly 290,000 people-are food insecure (21 per cent moderately and 15 per cent severely food insecure) [7]. Soaring of food prices [7] and waning of tourism due to the restrictions has further deteriorated the situation. The need to lessen economic effects of the pandemic was recognized by the government thus launching a stimulus package worth of N$8.1 billion aimed at assisting the individuals who lost their jobs including businesses and households, and payment of services rendered to the government [8]. However, this hardly compensated the economic downturn caused by the restrictions. The country continues to face various challenges in addressing the pandemic. There is a shortage of facilities, insufficient personal protective equipment (134 health workers have been infected as of 16 August 2020), shortage of testing kits and transport medium [3] (39187 out of 2.5 million population [9] tested as of 16 August 2020 [3].), inadequate ICU equipment, and lack of medical personnel [3]. The number of people in isolation who need medical attention is high thus creating a demand for medical doctors.

The number of confirmed cases were 203 with zero death on 29 June 2020. New cases started to rise and reached to 3101 on 11 August 2020, after the country entered Stage Four in which many unessential businesses resumed [3]. The people do not follow the restrictions as seriously as they did during the early days of the epidemic, as they need to seek for jobs and provisions outside, thereby contributing to the spread and emergence of new cases. Following the surge of new cases, on 31 July when the country was in Stage Four of state emergency, the government decided to tighten the restrictions with effectivity on 4 August for 28 days [4]. Based on this policy, schools were closed for the second time in four months, and public gatherings decreased from 250 to 100 individuals. People were only allowed to consume alcohol at home, and not in bars and taverns. Tourists are not required to pass a 14-day quarantine on their arrival, but should have a negative polymerase chain reaction (PCR) test taken 72 hours before their arrival. The country could not re-impose a full lockdown due its huge economic costs [4]. The country was reverted to stage 3 on 12 August and travel restrictions were imposed in some regions due exacerbation of the situation [3]. The current isolation guideline in Namibia is, as per the new directives of WHO, updated on 27 May 2020. The period of isolation is now 10 days plus at least three extra days from the last symptom for patients who had previously been symptomatic. Release from isolation is also now based on the number of days spent in isolation and not necessarily on a negative swab, however, if PCR tests are to be considered, it is still as per the old guidelines [6,10].

## Conclusion

The initiatives of Namibia in curbing the spread of COVID-19 has been calculated and nuanced. Policies implemented vary in response to the changing situation. Success of these policies can be achieved, however, through cooperation of the general public in following the guidelines recommended by the experts and devising timely and comprehensive strategies by the responsible authorities until a vaccine is available to all. The government should be more calculating and prudent when lifting restrictions to avoid its experiences of new case explosions. There should be a balance between the severity of the outbreak and the economic implications of restrictions when deciding on lockdowns. A second wave of the contagion is a possibility and may overburden the Namibian health system. Thus, the government needs to further strengthen its efforts particularly in equipping its health workers, increasing the facilities, improving on testing, tracing and treating, increasing health manpower, assisting the affected population economically, and invigorating public health campaigns.

## References

[1] Lucero-Prisno III DE, Ahmadi A, Essar MY, Lin Xu, Adebisi YA (2020). Addressing COVID-19 in Afghanistan: What are the efforts and challenges?. J Glob Health.

[2] Worldometer Namibia: Coronavirus Updates

[3] WHO Africa Namibia COVID-19 Situation Reports.

[4] U.S NEWS Namibia to Close Schools, Limit Public Gatherings as COVID-19 Cases Surge.

[5] Lucero-Prisno DE, Adebisi YA, Lin X (2020). Current efforts and challenges facing responses to 2019 nCoV in Africa. Glob Health Res Policy.

[6] Ministry of Health and Social Services of Namibia (2020). Information on COVID-19 pandemic.

[7] OCHA SADC RVAA (2019). Namibia: Vulnerability Assessment Committee Results.

[8] Deloitte COVID-19 Pandemic: Announcement of economic stimulus and relief package by the Minister of Finance.

[9] Worldometer Namibia population.

[10] WHO Criteria for releasing COVID-19 patients from isolation.

